# Psychosocial correlates of dietary fat intake in African-American adults: a cross-sectional study

**DOI:** 10.1186/1475-2891-8-15

**Published:** 2009-03-25

**Authors:** Joanne L Watters, Jessie A Satia

**Affiliations:** 1 Cancer Prevention Fellowship Program, Office of Preventive Oncology, National Cancer Institute, National Institutes of Health, Bethesda, Maryland 20852, USA; 2 Department of Nutrition, University of North Carolina at Chapel Hill, Chapel Hill, NC 27599, USA; 3 Department of Epidemiology, University of North Carolina at Chapel Hill, Chapel Hill, NC 27599, USA; 4 Lineberger Comprehensive Cancer Center, University of North Carolina at Chapel Hill, Chapel Hill, NC 27599, USA; 5 Center for Gastrointestinal Biology and Disease, Division of Digestive Diseases and Nutrition, University of North Carolina at Chapel Hill, Chapel Hill, NC 27599, USA

## Abstract

**Background:**

Current dietary guidelines recommend that dietary fat should comprise 20–35% percent of total energy intake, with less than 10% of energy from saturated fat. However, many Americans exceed these goals and data suggest that African Americans tend to consume a higher percentage of energy from dietary fat than Whites. Because diets low in dietary fat, particularly saturated fat, are associated with lower risk for many chronic illnesses, it is important to identify strategies to reduce high fat intakes. This study examined associations of psychosocial factors with dietary fat intake in African American adults 18 to 70 years.

**Methods:**

Data are self-reported from a cross-sectional survey of African Americans (n = 658) using an 11-page questionnaire, collected from June to October 2003. Associations of psychosocial (predisposing, reinforcing, and enabling) factors based on the PRECEDE framework, dietary fat-related behaviors, and participant characteristics (e.g., age, sex, education, BMI) with total and saturated fat consumption are described using linear regression and analysis of variance.

**Results:**

The mean age of participants was 43.9 years, 57% were female, 37% were college graduates, and 76% were overweight/obese. Respondents with lower fat intakes were female, older, had high education and very good/excellent perceived health. Among the psychosocial factors, the strongest (inverse) associations with fat intake were with two predisposing factors: *belief in the importance of a low-fat diet *(both genders) and *high self-efficacy *(women only). Fat intake was also significantly lower among participants who could *count on those close for encouragement to eat healthy foods *(a reinforcing factor) and among men who *needed more information about preparing healthy foods *(an enabling factor).

**Conclusion:**

Dietary interventions to decrease fat intake in African American adults may benefit from incorporating predisposing factors, such as personal beliefs and self-efficacy, in their design and implementation.

## Background

Diets low in dietary fat, particularly saturated fat, are associated with lower risk for obesity and many chronic illnesses [1,2]. African Americans have a disproportionately higher burden of many diet-related chronic diseases, including diabetes, cardiovascular disease, and many cancers [3,4]. Current dietary guidelines recommend that 20–35% percent of total energy intake comes from fat (<10% of energy from saturated fat) [5], yet African Americans tend to exceed these guidelines more often than Whites [6-9]. For example, based on the 1994–1996 USDA's Continuing Survey of Food Intakes by Individuals (CSFII), only 25% of non-Hispanic Blacks met dietary fat recommendations [10]. Furthermore, approximately 70% of African Americans are overweight or obese, considerably higher than the national average of 57% [11]. However, obesity likely results from a complex combination of environmental, metabolic, and genetic factors and a diet low in fat is not sufficient to prevent obesity.

Results of interventions to decrease fat consumption in the general population have been mixed, with an average reduction in percent energy from fat of seven percent [12]. Most of these dietary change programs have incorporated demographic characteristics, such as age, sex, education, physical activity, and socioeconomic status, as these factors have been associated with higher fat intake in African Americans. Psychosocial factors, such as diet-related knowledge, attitudes, beliefs, self-efficacy, and social support, have also been shown to play an important role in dietary behavior. For example, results from the National Cancer Institute's Five A Day program showed that psychosocial factors were more important determinants of fruit and vegetable intake than demographic factors alone [13]. However, few studies have examined relationships between psychosocial factors and fat intake in African Americans. Given that psychosocial factors, along with demographic and environmental characteristics, are part of the complex interplay that affects food choices, better understanding of how these variables may affect fat intake could be useful in designing more effective interventions for African Americans.

The PRECEDE (Predisposing, Reinforcing, and Enabling Constructs in Educational Diagnosis and Evaluation) framework, which has been used to understand motivations for healthy dietary behaviors and mediating factors in dietary interventions, categorizes psychosocial variables into 3 main categories: predisposing, reinforcing, and enabling factors [14]. It is important to note that while PRECEDE serves as framework for organizing factors and identifying intervention strategies, it does not predict or explain these factors [15]. Predisposing factors are antecedents that influence the likelihood of how one will behave and include the individuals' knowledge, attitudes, beliefs, and self-efficacy [15]. Reinforcing factors are incentives following a behavior that may affect the likelihood that this behavior will be repeated over time, such as social support, peer influence, and rewards [15]. Enabling factors help facilitate behavior and include programs, services, and resources necessary for a behavior to occur [15]. PRECEDE is considered a particularly suitable conceptual framework for studies of minority populations because it is adaptable to the population of interest [15].

The objective of these analyses is to assess associations of psychosocial factors with intakes of total and saturated fat in a sample of African American men and women. Given the high rates of fat-related medical conditions in African Americans, this work has important applications for dietary interventions designed to decrease fat consumption in African Americans.

## Methods

### Study Design and Population

Data presented here were collected for a study examining methods and strategies to recruit African Americans into cancer prevention studies. Details on the study design and data collection procedures are described elsewhere [16]. Briefly, 5,000 potential African American participants residing in six North Carolina counties (three urban, three rural), 18 to 70 years, were randomly selected from Department of Motor Vehicle rosters. Prospective participants were asked to complete a questionnaire for a study examining diet and health in African Americans. Participants were informed that all responses would be kept confidential and anonymous. One of five different recruitment strategies, based on variations of approach letters (generic vs. culturally sensitive) and type of incentive (included, excluded, or promised), were randomly assigned to each participant. All prospective participants were sent a questionnaire by mail with a prepaid return envelope and instructions for completing the survey via the Internet or by a toll-free telephone line. The study response rate was 17.5% (n = 747): 87.7% by mail, 11.2% via the Internet, and 1.1% by telephone and response rates were higher for the strategies that included incentives than for those that did not; response rates were 24%, 16%, and 19% for included, excluded, or promised incentive, respectively. Data were excluded from 89 respondents who did not meet age and county eligibility criteria or with questionnaires that failed quality-control checks, leaving 658 persons for these analyses. The study received human subjects research approval and was compliant with HIPAA guidelines.

### Data Collection

Using the PRECEDE planning model as a guide [14,15,17], an 11-page questionnaire was created to capture demographic, psychosocial, lifestyle, and behavioral factors related to cancer prevention. The questionnaire was pretested in a small convenience sample and questions were adapted from those that have been used in other studies [18-20], whenever possible. Four sets of questions were used in these analyses: demographic characteristics, diet-related psychosocial factors, fat-related diet habits, and dietary fat intake. Data were collected from June to October 2003 and analyzed in 2006 and are from self-report.

### Psychosocial factors

*Predisposing factors *included questions regarding *knowledge *– whether participants had heard about the U.S. Department of Agriculture's Food Guide Pyramid (yes, no, don't know/not sure); *attitudes *– whether they believe a diet and cancer relationship exists, and if so, whether the relationship is strong, moderate, or weak, and how important it is for them personally to eat a diet low in fat (very, somewhat, or not important); and *self-efficacy*. Healthful eating self-efficacy was assessed by a Likert-scale item about respondents' confidence (very confident, somewhat confident, or not confident) in their ability to eat less fat.

#### Reinforcing factors addressed social support

Respondents were asked whether they felt they could count on those close to them: to encourage them to eat healthfully; to tell them about healthier foods and how to prepare them; to prepare healthier foods with them; and to eat healthier foods with them. Response options were a lot, some, or not at all.

*Enabling factors *included four items related to *perceived barriers to healthy eating *and queried respondents on whether: they can afford to purchase healthy foods and meals; it takes too much time and trouble to prepare healthy meals, it is easy for them to order healthy foods in restaurants; and they need more information on how to prepare healthy foods and meals. Possible responses were yes, sometimes, or no.

For each set of factors, scales were created by summing responses to individual questions (least healthy responses scored the lowest and the healthiest responses scored the highest). A summary score for each scale was computed as the mean of the non-missing responses.

### Assessment of fat intake and fat-related diet habits

Intakes of total fat and saturated fat "over the past 3 months" were captured using a fat screener developed by Block and colleagues, which is composed of 13 items designed to capture common sources of fat in the American diet [21,22]. To obtain mean daily intakes, we followed the Block algorithm and multiplied the number of times each item was consumed by the reported age- and sex-specific portion size and summed across the daily intakes for all 13 items. Because the Block fat screener assesses intakes of only 13 food items, it does not measure total fat consumption. Rather, it provides an estimate of fat intake suitable for ranking purposes.

Fat-related dietary habits were captured using a slightly modified version of the Fat-Related Diet Habits Questionnaire, a 12-item instrument that assesses the following fat-related behaviors: avoiding fat as a flavoring, substituting specially manufactured low-fat foods, modifying meats to be lower in fat, replacing high-fat foods with fruits and vegetables, and avoiding fried foods. Possible responses ("usually," "often," "sometimes," or "rarely/never") were coded one to four, where a low score corresponded to lower fat intake. The summary score was calculated as the mean of the non-missing items. Good face validity, internal consistency, and modest test-retest correlations were reported for this instrument [23,24].

### Demographic characteristics

Various demographic characteristics were assessed, including age (categorized approximately into tertiles), sex, education (less than or equivalent to high school, some college, college graduate, or advanced degree), marital status (never married, married/living with partner, or divorced/separated/widowed), and perceived health status (excellent, very good, good, fair, or poor). Using self-reported height and weight, body mass index (BMI) was calculated as kg/m^2 ^and categorized as normal (18.5 to 24.9), overweight (25.0 to 29.9), and obese (≥ 30.0) [25].

### Statistical Analyses

Data analyses were conducted using Stata (version SE 8.2, STATACorp, College Station, TX). Descriptive statistics (means and percentages) were calculated for all demographic and psychosocial variables. For each demographic characteristic, one-way ANOVA models were used to determine whether there were statistically significant differences between the mean values of each psychosocial (i.e., predisposing, reinforcing, and enabling) scale score, fat-related diet habits scale score, and mean daily total fat and saturated fat intake (grams/day). Multiple linear regression was used to assess associations of the psychosocial scales (categorized into approximate tertiles) with total fat and saturated fat intakes, unadjusted and adjusted for age, sex, education, and BMI. Relationships between each individual psychosocial factor (categorized by least healthy to most healthy response) and fat consumption were also examined using multiple linear regression models, unadjusted and adjusted for participant characteristics and the other predisposing, reinforcing, and enabling factors. To examine potential multi-colinearity, we computed variance inflation factors (VIF) for all predisposing, reinforcing, and enabling factors (separately and in a single model) and all VIFs were well below 10. *p *values were computed to test for statistically significant overall differences in means. The dietary variables were not transformed because the data were not markedly skewed. Statistical tests were two-sided and *p *values ≤ 0.05 were considered statistically significant.

## Results

The distribution of responses to each psychosocial (predisposing, reinforcing, and enabling) factor for the study sample is given in Table 1. About a third of respondents believed that it is *very important to eat a diet low in fat *and 44% were very confident that they had the ability *(self-efficacy) *to eat less fat. Most felt they could count on those close to them "some" *to tell them about healthier foods, prepare healthier foods with them*, and *eat healthy foods *and 48% felt they could count on those close to them "a lot" *to encourage them to eat healthy foods*. The majority of respondents *could afford to purchase healthy foods *and *needed more information on how to prepare healthy foods*.

**Table 1:** Distribution of participants by response to each psychosocial factor (n = 658)^1^

	**Healthiest** **Response**	**N** **(%)**	**Moderate** **Response**	**N** **(%)**	**Least Healthy Response**	**N** **(%)**
**Predisposing Factors**						
Do you think what you eat and drink are related to your own chance of getting cancer? (Yes/No); Do you think this relationship between diet and cancer is:	Yes, Strong	324 (49%)	Yes, Moderate	198 (30%)	Yes, Weak Or No	136 (21%)
How important is it to you personally to eat a diet low in fat?	Very Important	222 (34%)	Somewhat Important	309 (48%)	Not Important	118 (18%)
If you wanted to eat less fat, how confident are you that you could do it?	Very Confident	285 (44%)	Somewhat Confident	280 (43%)	Not Confident	81 (13%)
Have you ever heard of the Food Guide Pyramid?	Yes	533 (82%)	Not Sure/Don't Know	94 (14%)	No	25 (4%)
						
**Reinforcing Factors**						
If you tried to eat healthier foods, how much could you count on the people close to you to:						
Encourage you.	A lot	310 (48%)	Some	261 (40%)	Not at all	76 (12%)
Tell you about healthier foods and how to prepare them.	A lot	164 (26%)	Some	336 (52%)	Not at all	142 (22%)
Prepare healthier foods with or for you.	A lot	161 (25%)	Some	300 (46%)	Not at all	185 (29%)
Eat healthier foods with you.	A lot	198 (31%)	Some	361 (56%)	Not at all	89 (14%)
						
**Enabling Factors**						
Do you feel that you can afford to purchase healthy foods?	Yes	463 (72%)	Sometimes	127 (20%)	No	55 (9%)
Do you feel that it takes a lot of time and trouble to prepare healthy foods and meals?	No	338 (52%)	Sometimes	146 (23%)	Yes	162 (25%)
Do you feel that it is easy for you to order healthy foods when you go out to eat at restaurants?	Yes	246 (38%)	Sometimes	205 (32%)	No	196 (30%)
Do you more need information on how to prepare healthy foods and meals?	No	196 (30%)	Sometimes	75 (11%)	Yes	379 (58%)

^1^Numbers may not add up to 658 and percentages may not add up to 100% due to rounding and missing data

Table 2 gives mean psychosocial scale scores, fat-related diet habit scale scores, and total and saturated fat intakes by participant demographic characteristics. The mean age was 43.9 years, 57% were female, 77% had some college education or higher, 76% were overweight or obese, 56% were married/living with partner, and 43% had excellent or very good perceived health status. Compared to males, females had significantly lower enabling factor scores and lower total and saturated fat intakes. Older participants reported higher predisposing, reinforcing, and enabling scores and lower total and saturated fat intakes than younger participants. High educational level was positively associated with predisposing scale scores and inversely correlated with saturated fat intake. Respondents with excellent/very good perceived health had higher predisposing, reinforcing, and enabling scale scores and lower total and saturated fat consumption than those with fair/poor perceived health. Similar results were observed using the fat-related diet habits scale: older participants, those with more formal education, and those with excellent self-reported health reported lower fat-related diet habit scores, which corresponds to lower fat consumption.

**Table 2:** Mean psychosocial scale scores and fat intakes by participant characteristics (n = 658)

		**Mean Scale Score^1^**			**Fat Screener (g/day)^2^**		
**Characteristic**	**N (%)^4^**	**Predisposing**	**Reinforcing**	**Enabling**	**Total Fat**	**Saturated Fat**	**Fat-related diet habits scale score^3^**
**Sex**							
Male	271 (41%)	2.23	2.24	2.23	33.5	12.8	2.92
Female	378 (57%)	2.26	2.05	2.13	30.0	11.1	2.93
Overall *p *value		0.42	0.09	0.01	0.09	0.008	0.59
**Age (years)**							
20–34	154 (23%)	2.16	2.04	2.13	38.8	15.1	2.95
35–49	286 (43%)	2.25	2.15	2.13	31.5	11.7	2.96
50–70	218 (33%)	2.30	2.17	2.26	26.1	9.6	2.87
*p *for trend		0.005	0.01	<0.001	0.01	<0.001	0.03
**Education**							
< High School	146 (23%)	2.11	2.06	2.15	37.2	13.9	3.01
Some College	256 (40%)	2.22	2.13	2.14	31.3	11.7	2.94
College graduate	168 (26%)	2.31	2.17	2.22	27.9	10.7	2.88
Advanced Degree	74 (11%)	2.45	2.15	2.23	28.7	10.6	2.83
Overall *p *value		<0.001	0.69	0.26	0.69	<0.001	<0.001
**Body Mass Index (kg/m^2^)**							
Normal (18.5–24.9)	151 (24%)	2.34	2.16	2.08	32.8	12.6	2.99
Overweight (25–29.9)	227 (35%)	2.41	2.11	2.04	29.5	11.1	2.91
Obese (≥ 30)	266 (41%)	2.36	2.13	2.01	32.8	12.2	2.91
*p *for trend		0.97	0.48	0.08	0.84	0.85	0.04
**Marital Status**							
Single	177 (27%)	2.23	1.99	2.11	34.7	13.2	2.94
Married/Living with partner	368 (56%)	2.45	2.22	2.22	30.2	11.4	2.92
Separated or Divorced	88 (13%)	2.23	2.01	2.11	30.8	11.3	2.94
Widowed	19 (3%)	2.38	2.28	2.10	26.8	9.2	2.76
Overall *p *value		0.45	0.21	0.03	0.21	0.03	0.54
**Perceived Health Status**							
Excellent	67 (10%)	2.34	2.23	2.28	30.9	12.0	2.83
Very Good	214 (33%)	2.30	2.13	2.24	28.3	10.6	2.90
Good	260 (40%)	2.22	2.12	2.14	31.4	11.8	2.93
Fair	93 (14%)	2.17	2.08	2.04	37.0	13.9	3.03
Poor	13 (2%)	2.04	2.23	2.15	50.3	17.8	2.91
Overall *p *value	0.002	0.004	0.004	0.004	0.001	0.02	

^1^Psychosocial scales were created by combining responses to individual questions (least healthy responses scored the lowest and the healthiest responses scored the highest).

^2 ^Fat intake was estimated using 13-item Block fat screener.

^3^The fat-related diet habits scale score was calculated using responses to 12 items about dietary behaviors; a higher number corresponds to higher fat intake.

^4^Numbers may not add up to 658 and percentages may not add up to 100% due to rounding and missing data.

The mean total and saturated fat intake and mean fat-related diet habits scale score are given for each individual predisposing, enabling, and reinforcing factor in Additional file 1, Table S1. All analyses were adjusted for age, sex, education, BMI, and the other psychosocial factors within that category (i.e., predisposing, reinforcing, or enabling). Two of the four predisposing factors were significantly associated with lower total and saturated fat intake and lower fat-related diet habits: *belief in the importance of eating a diet low in fat *and *high self-efficacy to eat less fat*. Respondents who *believed a low-fat diet is very important *consumed a mean of 27.5 g/day of total fat and 10.3 g/day of saturated fat, compared to intakes of 38.3 g/day and 14.3 g/day, respectively, among those who did not share this belief. Similarly, those with high self-efficacy reported consuming approximately 6 g/day of total fat and 2 g/day of saturated fat less than respondents with low self-efficacy. One reinforcing factor, *can count on those close to you to encourage you to eat healthy foods*, was significantly associated with fat consumption, but not the fat-related diet habits. Participants who felt they could count on those close to them "a lot" consumed approximately 1 g/day more total and saturated fat than those who responded "not at all" (p = 0.03). Among the enabling factors, the only variable statistically significantly associated with fat consumption was *need for information on how to prepare healthy foods*; participants who needed additional information consumed 6.0 g/day of total fat and 2.1 g/day of saturated fat less than those who felt they did not need more information. The amount of variance explained by the demographic and psychosocial factors ranged from 2% (adjusted R^2 ^for reinforcing and enabling factors for fat-related habits) to 17% (unadjusted R^2 ^for predisposing factors for saturated fat); only 2–3% of the variance was explained by demographic variables alone. There was little difference in the amount of variance explained between models that included all predisposing, reinforcing, and enabling factors together and predisposing factors separately.

Additional file 1, Table S2 gives associations of each significant individual psychosocial factor from Additional file 1, Table S1 with fat consumption and the fat-related diet habits scale in the total study population and stratified by sex. After controlling for age, education, BMI, and all other significant psychosocial factors, *belief in the importance of a low-fat diet *and *high self-efficacy to eat less fat *were each associated with significantly lower total and saturated fat intakes in the combined sample. However, in stratified analyses, *belief in the importance of a low-fat diet *and *high self-efficacy to eat less fat *were only statistically significant for women, whereas men who *needed more information on how to prepare healthy foods *consumed less total fat than those who believed they had sufficient information. Results were generally similar for the diet habits scale, except that men who *believed in the importance of a low-fat diet *and had *high self-efficacy to eat less fat *had significantly lower fat-related diet habits scores. Respondents who *could count on those close to them to encourage them to eat healthy foods *consumed less total fat than those who did not have such support (p = 0.05), which is a reversal of the association reported in Additional file 1, Table S1. There were no statistically significant associations for any reinforcing or enabling factors with the fat-related diet habits in Additional file 1, Table S1.

## Discussion

This study examined psychosocial correlates of total and saturated fat intake and fat-related dietary habits, using the PRECEDE framework, in a sample of 658 African American men and women in North Carolina. The two psychosocial factors most strongly inversely associated with total and saturated fat intake were predisposing factors: *strong belief in the importance of a low-fat diet *and *high self-efficacy to eat less fat*. One enabling factor, *need for information on how to prepare healthy foods*, was inversely associated with total and saturated fat consumption in men only, while *could count on those close to you to encourage you to eat healthy foods *(a reinforcing factor) had a weak inverse relationship with total fat intake in the combined sample.

Several demographic characteristics, specifically age, education, and perceived health status, were associated with the psychosocial scales, fat-related dietary behaviors, and/or fat intake. Older respondents and those with very good/excellent perceived health had higher predisposing, reinforcing, and enabling scores. Highly educated participants also reported higher predisposing scores, whereas enabling scale scores were higher in males. Respondents who were younger (20–34 years), male, unmarried, and who had fair/poor perceived health had higher saturated fat intakes. These findings are in agreement with those reported in other studies of African Americans [7,8,26]. For example, in a survey of 2,172 African Americans in North Carolina, saturated fat intakes were higher in men and those with less education and, in contrast with our results, also higher among those with better perceived health and 50 years of age or older [8]. Baseline data from the Working Well Trial similarly found that younger participants and those with higher education had lower fat intakes [27,28]. The association between higher levels of education and lower fat intake was also reported in a study of urban low, income African Americans [26]. Fat-related dietary behavior scale scores were higher for those with BMIs <25 kg/m^2 ^than for those who were overweight or obese; however, there were no associations for BMI with fat intake or any psychosocial factor. The role of dietary fat in weight loss has been widely debated [29,30] and is beyond the scope of this study, our purpose is solely to examine associations of dietary fat intake as part of a generally healthy diet, not to make inferences about weight loss or maintenance.

The greatest impact on fat intake and fat-related dietary habits was seen for the predisposing factors: *belief in the importance of eating a low-fat diet *and *self-efficacy to eat less fat*. Those who felt a low-fat diet was important consumed less total and saturated fat and scored lower on the fat-related diet habits scale than those who felt it was not important. Similarly, respondents who were very confident in their ability to eat less fat consumed less fat than those with low self-efficacy (Additional file 1, Table S2). These strong associations suggest that dietary interventions would benefit from incorporating these two predisposing factors in their design, particularly since sizeable numbers of study participants reported "less healthy" responses for these key correlates of fat intake: only 34% believed it was "very important" to eat a low-fat diet and less than half (44%) felt they were very confident they could eat less fat. High self-efficacy has consistently been shown to influence healthy eating behaviors, such as higher fruit and vegetable intakes, in other studies [13,31-33].

In sex-stratified analyses of the associations of the psychosocial factors with fat consumption, both *belief in the importance of a low-fat diet *and *self-efficacy *were strongly associated with total and saturated fat consumption in women, but not men. Although both factors were statistically significantly associated with lower fat-related diet habits scores in men. To our knowledge, self-efficacy to eat a low-fat diet has not been examined separately for African American men and women in previous studies. One possible reason why self-efficacy may be more salient for women than men is that women are more often responsible for food preparation.

One enabling factor, *need for more information on how to prepare healthy foods*, was inversely associated with fat intake in men; total fat intake was 26% higher among men who did not express this need relative to those who did. These results were somewhat unexpected, as we had hypothesized that respondents who believed that they had adequate information would apply this knowledge in preparing and consuming lower fat meals. We postulate several possible explanations for this finding. It is possible that participants may have responded that they did *not *need more information because they: 1) mistakenly believed they possessed all necessary information, 2) did not value a diet low in fat and therefore, desired no additional information, or 3) may have all the information necessary to consume a lower fat diet, may have varying levels of receptivity in applying their personal knowledge [34]. It is also plausible that, as written, the question did not capture what was intended, which is simply whether or not the participant had adequate information to prepare and eat healthy foods (i.e., foods low in fat).

None of the reinforcing factors were significantly associated with fat consumption in either men or women separately. In the combined sample, there was a weak association of total fat with *can count on those close encourage you to eat healthy foods*. This result is somewhat surprising, as social support has been found to be associated with dietary change [35] and preventive health practices [36] in African Americans in some, but not all studies [37]. It is possible that the specific social support variables measured here may not be salient for this population with regards to fat intake.

Psychosocial factors, particularly predisposing factors, have been associated with fat intake and fat-related dietary behaviors in other studies designed on the PRECEDE framework [17,18,38]. In the Working Well Trial, Kristal et al. also found that predisposing factors were stronger predictors of fat intake than reinforcing or enabling factors in a largely White population; participants with the highest predisposing scores consumed approximately 29% less total fat than those with the lowest predisposing scores [18]. Predisposing factors were also more strongly associated with fat intake than enabling factors in a study of predominantly White male auto workers [17]. Unfortunately, because these studies did not include adequate numbers of African Americans, we are unable to directly compare our results. Furthermore, very few studies of African American men and women have examined relationships of psychosocial factors with fat intake. One study [37] of 850 African Americans failed to show a relationship between social support and fat consumption, which mirrors the absence of an association between reinforcing factors and fat intake in this report.

This study has a number of strengths. This is among the first studies that examined psychosocial, as well as demographic and lifestyle, factors related to fat consumption in African Americans. The sample size was large enough (n = 658) to allow detection of associations that may be obscured in smaller studies. Also, questions in our survey instrument designed to capture psychosocial factors were adapted from previous studies that used the PRECEDE framework to examine psychosocial variables as mediating factors in healthy eating interventions [18-20].

Our study also has some limitations. First, the overall response rate was relatively low (17.5%), which may limit the generalizability of our findings and we are unable to compare responders and non-responders with regard to demographic and other characteristics. Based on 2000 US Census data for the six counties included in this study and NC state data in the Behavioral Risk Factor Surveillance Survey (BRFSS), our sample is generally comparable to African Americans in NC [5,39]. In addition, persons who are willing to participate in a research study about diet and health may be more conscious about health, diet, and fat intake than the general public. Second, all data are from self-report, which is subject to both random and systematic bias [40]. Third, total and saturated fat intake were underestimates of actual daily intake because the instrument used here consists of only 13 food items [41]. It is possible these results may be affected by differences in fat intake from foods not reflected in the 13-item fat screener; however, both the fat screener and the fat-related diet habits questionnaire have been validated against longer food frequency questionnaires in multi-racial and ethnic populations [23,24,41]. Fourth, most of the psychosocial factors were assessed via single item measures; therefore, all the components of complex concepts such as social support were likely not captured. In addition, it is possible that the questionnaire may not have accurately captured all important factors affecting fat intake. Future investigations may benefit from employing a broader model of health behavior with additional constructs, e.g., Social Cognitive Theory, and more detailed assessment tools. Fifth, because this is a cross-sectional study, changes in psychosocial factors were not compared with increases in fat intake; hence causality should not be inferred. Furthermore, it is not possible to determine whether fat intakes drive psychosocial factors or vice-versa or whether additional other unmeasured factors could be responsible for differences in fat intakes. Finally, it is worth noting that during the time these data were collected, high-fat, low-carbohydrate diets were popular, which may have affected perceptions about dietary fat.

In summary, many healthy eating interventions target reinforcing (social support) and enabling (barriers) factors. However, based on the results of this study, interventions that target predisposing factors, specifically personal beliefs and self-efficacy, may be more successful. This does not mean that reinforcing and enabling factors should be excluded from healthy eating interventions, but rather, that predisposing factors may have a greater impact on fat intake and should therefore be emphasized. Participants consumed lower amounts of total and saturated fat if they *believed that a low-fat diet is important*. Women consumed less fat if they had high *self-efficacy*, whereas men who *needed more information on how to prepare healthy foods *consumed higher amounts of fat. Based on these results, optimal interventions to decrease total and saturated fat intakes in African American adults should target increasing participants' *belief in the merits of a low-fat diet*. Men in such programs may benefit from the *provision of information on how to prepare foods that are low in fat*, and for women, *healthy eating self-efficacy *should be encouraged.

## Conclusion

Based on the results of this study, predisposing factors, specifically personal beliefs and self-efficacy, were the psychosocial factors most strongly associated with fat intake in African American adults. Thus, healthy eating interventions designed to impact fat intake benefit from emphasizing such predisposing factors, especially for those programs targeting African Americans.

## Competing interests

The authors declare that they have no competing interests.

## Authors' contributions

Both authors significantly contributed to the preparation of this manuscript and have approved the final version. JS led the conception and design of the study. JW was primarily responsible for the analysis and interpretation of the data.

## Supplementary Material

Additional file 1 **Table S1 and Table S2.** Table S1 – Adjusted mean fat intake and fat behavior scale scores by individual psychosocial factors (n = 658). Table S2 – Adjusted mean fat intake by all significant psychosocial factors (n = 658). Click here for file
