# Supplementary material for: Psychosocial correlates of dietary fat intake in African-American adults: a cross-sectional study
Source: Nutr J. 2009 Mar 25;8:15. doi: 10.1186/1475-2891-8-15 (PMC2667442; doi:10.1186/1475-2891-8-15)
Supplement: Additional file 1 — Table S1 and Table S2. Table S1 – Adjusted mean fat intake and fat behavior scale scores by individual psychosocial factors (n = 658). Table S2 – Adjusted mean fat intake by all significant psychosocial factors (n = 658). [file 1475-2891-8-15-S1.doc]

**Table S1 -** Adjusted[[1]](#footnote-2) mean fat intake and fat behavior scale scores by individual psychosocial factors (n=658)

|  | **Predisposing Factors** | | | |  |  | **Reinforcing Factors** | | | |  |  | **Enabling Factors** | | | |  | |
| --- | --- | --- | --- | --- | --- | --- | --- | --- | --- | --- | --- | --- | --- | --- | --- | --- | --- | --- |
| Responses[[2]](#footnote-3) | Belief that your diet is related to your cancer risk | Belief in importance of a low-fat diet | Self-efficacy to eat  less fat | Aware  of Food Guide Pyramid | Unadj. R^2^ | Adj. R^2^ | Can you count on those close to you to: | | | | Unadj. R^2^ | Adj. R^2^ | Feel  you can afford healthy foods | Time/  trouble to prepare healthy foods | It is easy to order healthy foods at restaurants | Need information to prepare healthy foods | Unadj. R^2^ | Adj. R^2^ |
|  |  |  |  |  |  |  | Encourage you to eat healthy foods | Tell you about healthier foods | Prepare healthier foods with you | Eat healthier foods with you |  |  |  |  |  |  |  |  |
| **Fat Screener[[3]](#footnote-4): Total Fat (g/day)** | | |  |  | 16% | 15% |  |  |  |  | 12% | 10% |  |  |  |  | 13% | 11% |
| Healthiest | 33.3 | 27.5 | 29.3 | 32.3 |  |  | 33.9 | 29.2 | 30.0 | 32.2 |  |  | 30.9 | 31.5 | 32.0 | 35.3 |  |  |
| Moderate | 29.9 | 32.2 | 33.1 | 29.8 |  |  | 28.6 | 31.8 | 33.0 | 31.1 |  |  | 32.4 | 30.8 | 29.0 | 34.1 |  |  |
| Least Healthy | 32.3 | 38.3 | 35.4 | 26.8 |  |  | 33.0 | 34.1 | 31.0 | 32.8 |  |  | 36.3 | 32.6 | 33.9 | 29.3 |  |  |
| *p* value | 0.28 | <0.001 | 0.03 | 0.3 |  |  | 0.03 | 0.36 | 0.46 | 0.79 |  |  | 0.19 | 0.76 | 0.06 | 0.004 |  |  |
|  |  |  |  |  |  |  |  |  |  |  |  |  |  |  |  |  |  |  |
| **Fat Screener: Saturated Fat (g/day)** | | | |  | 17% | 15% |  |  |  |  | 14% | 12% |  |  |  |  | 14% | 12% |
| Healthiest | 12.5 | 10.3 | 11.1 | 12.1 |  |  | 12.9 | 10.8 | 11.2 | 12.1 |  |  | 11.6 | 12.0 | 12.1 | 13.2 |  |  |
| Moderate | 11.2 | 12.2 | 12.3 | 11.3 |  |  | 10.7 | 12.0 | 12.4 | 11.7 |  |  | 12.1 | 11.5 | 10.9 | 13.0 |  |  |
| Least Healthy | 12.1 | 14.3 | 13.4 | 10.6 |  |  | 12.0 | 12.9 | 11.7 | 12.4 |  |  | 13.8 | 12.1 | 12.6 | 11.1 |  |  |
| *p* value | 0.28 | <0.001 | 0.04 | 0.48 |  |  | 0.02 | 0.29 | 0.48 | 0.73 |  |  | 0.16 | 0.82 | 0.1 | 0.008 |  |  |
|  |  |  |  |  |  |  |  |  |  |  |  |  |  |  |  |  |  |  |
| **Fat-related diet habits scale score[[4]](#footnote-5)** | | | | | 17% | 15% |  |  |  |  | 4% | 2% |  |  |  |  | 4% | 2% |
| Healthiest | 2.9 | 2.8 | 2.8 | 2.9 |  |  | 2.9 | 2.9 | 2.9 | 3.0 |  |  | 2.9 | 2.9 | 2.9 | 2.9 |  |  |
| Moderate | 2.9 | 3.0 | 3.0 | 2.9 |  |  | 2.9 | 2.9 | 2.9 | 2.9 |  |  | 2.9 | 2.9 | 2.9 | 2.9 |  |  |
| Least Healthy | 2.9 | 3.1 | 3.1 | 2.9 |  |  | 3.0 | 2.9 | 3.0 | 2.9 |  |  | 2.9 | 3.0 | 3.0 | 2.9 |  |  |
| *p* value | 0.72 | <0.001 | <0.001 | 0.99 |  |  | 0.61 | 0.63 | 0.40 | 0.82 |  |  | 0.99 | 0.47 | 0.45 | 0.97 |  |  |

**Table S2 -** Adjusted[[5]](#footnote-6) mean fat intake by all significant psychosocial factors (n=658)

|  | **Belief in importance of a**  **low-fat diet** | | |  | **Self-efficacy to eat less fat** | | |  | **Can count on those close to you to encourage you to**  **eat healthy foods** | | |  | **Need information on how to**  **prepare healthy foods** | | | **Adj. R^2^** | |
| --- | --- | --- | --- | --- | --- | --- | --- | --- | --- | --- | --- | --- | --- | --- | --- | --- | --- |
|  | Fat Screener**[[6]](#footnote-7)** | |  |  | Fat Screener | |  |  | Fat Screener | |  |  | Fat Screener | |  |  |  |
|  | Total  Fat (g/day) | Saturated Fat  (g/day) | Fat-Related Diet Habits Score**[[7]](#footnote-8)** |  | Total Fat (g/day) | Saturated Fat (g/day) | Fat-Related Diet Habits Score |  | Total  Fat (g/day) | Saturated  Fat  (g/day) | Fat-Related Diet Habits Score |  | Total Fat (g/day) | Saturated Fat (g/day) | Fat-Related Diet Habits Score | Total  Fat | Saturated Fat |
| **Men and Women** | |  |  | **Men and Women** | |  |  | **Men and Women** | | | | **Men and Women** | |  |  | 15% | 16% |
| Very Important | 27.9 | 10.5 | 2.8 | Very Confident | 29.1 | 11.0 | 2.8 | Yes | 33.0 | 12.4 | 2.9 | No | 34.2 | 12.8 | 2.9 |  |  |
| Somewhat Important | 32.1 | 12.1 | 3.0 | Somewhat Confident | 33.4 | 12.5 | 3.0 | Sometimes | 29.4 | 11.1 | 2.9 | Sometimes | 33.6 | 12.7 | 3.0 |  |  |
| Not Important | 37.9 | 14.2 | 3.1 | Not Confident | 35.2 | 13.4 | 3.1 | No | 34.8 | 12.9 | 3.0 | Yes | 30.1 | 11.3 | 2.9 |  |  |
| *p* value | <0.0001 | <0.0001 | <0.0001 | *p* value | 0.02 | 0.03 | <0.0001 | *p* value | 0.05 | 0.07 | 0.63 | *p* value | 0.06 | 0.08 | 0.34 |  |  |
|  |  |  |  |  |  |  |  |  |  |  |  |  |  |  |  |  |  |
| **Men Only** |  |  |  | **Men Only** |  |  |  | **Men Only** |  |  |  | **Men Only** |  |  |  | 10% | 12% |
| Very Important | 30.8 | 11.9 | 2.8 | Very Confident | 33.6 | 13.1 | 2.8 | Yes | 36.1 | 13.9 | 2.9 | No | 39.4 | 15.3 | 2.8 |  |  |
| Somewhat Important | 35.0 | 13.4 | 3.0 | Somewhat Confident | 35.3 | 13.3 | 3.0 | Sometimes | 32.0 | 12.4 | 2.9 | Sometimes | 38.9 | 15.5 | 3.0 |  |  |
| Not Important | 40.6 | 15.6 | 3.0 | Not Confident | 35.6 | 13.9 | 3.0 | No | 37.4 | 14.0 | 3.0 | Yes | 31.2 | 11.8 | 2.9 |  |  |
| p value | 0.08 | 0.10 | <0.0001 | *p* value | 0.83 | 0.91 | 0.02 | *p* value | 0.33 | 0.40 | 0.25 | *p* value | 0.02 | 0.008 | 0.13 |  |  |
|  | |  |  |  |  |  | |  |  |  |  |  | |  | |  |  |
| **Women Only** | |  |  | **Women Only** |  |  | | **Women Only** | |  | | **Women Only** | |  | | 18% | 19% |
| Very Important | 25.8 | 9.5 | 2.8 | Very Confident | 25.7 | 9.5 | 2.8 | Yes | 30.6 | 11.4 | 2.9 | No | 30.4 | 11.1 | 2.9 |  |  |
| Somewhat Important | 29.5 | 11.0 | 3.0 | Somewhat Confident | 31.7 | 11.7 | 3.0 | Sometimes | 27.3 | 10.1 | 2.9 | Sometimes | 29.6 | 10.8 | 2.9 |  |  |
| Not Important | 36.4 | 13.4 | 3.1 | Not Confident | 34.7 | 12.9 | 3.1 | No | 32.3 | 11.8 | 2.9 | Yes | 28.9 | 10.9 | 2.9 |  |  |
| p value | 0.003 | 0.005 | <0.0001 | *p* value | 0.004 | 0.004 | <0.0001 | *p* value | 0.14 | 0.16 | 0.79 | *p* value | 0.80 | 0.97 | 0.96 |  |  |

1. Mean values adjusted for all other factors within its category (i.e., predisposing, reinforcing, or enabling), age, sex, education, and BMI. [↑](#footnote-ref-2)
2. ?Responses in order of healthiest, moderate, and least healthy: **Predisposing factor*s***: *Belief in diet and cancer risk*: “Yes, strong”, “Yes, Moderate”, “Yes, Weak or No”; *Belief in importance of a low-fat diet*: “Very important,” “Somewhat important,” “Not Important”; *Self-efficacy*: “Very confident,” “Somewhat confident,” “Not confident”. **All Reinforcing factors***: “*A lot,” “Some,” “Not at all.” **Enabling factors***: Afford healthy foods*: “Yes,” “Sometimes,” “No”; *Takes time and trouble to prepare healthy foods*: “No,” “Sometimes,” “Yes”; *Easy to order healthy foods*: “Yes,” “Sometimes,” “No”; *Need information to prepare healthy foods*: “No,” “Sometimes,” “Yes”. [↑](#footnote-ref-3)
3. Fat intake was estimated using 13-item Block fat screener. [↑](#footnote-ref-4)
4. The fat-related diet habits scale score was calculated using responses to 12 items about dietary behaviors; a higher number corresponds to higher fat intake. [↑](#footnote-ref-5)
5. Mean values adjusted for all other factors deemed significant in Table S1, age, sex, education, and BMI. [↑](#footnote-ref-6)
6. Fat intake was estimated using 13-item Block fat screener. [↑](#footnote-ref-7)
7. The fat-related diet habits scale score was calculated using responses to 12 items about dietary behaviors; a higher number corresponds to higher fat intake. [↑](#footnote-ref-8)
